# Differential detection of tuberculous and non-tuberculous mycobacteria by qPCR in lavage fluids of tuberculosis-suspicious white rhinoceros

**DOI:** 10.1371/journal.pone.0207365

**Published:** 2018-11-28

**Authors:** Robert Hermes, Joseph Saragusty, Irmgard Moser, Stefanie A. Barth, Susanne Holtze, Alexis Lecu, Jonathan Cracknell, Duncan Williams, Frank Göritz, Thomas Bernd Hildebrandt

**Affiliations:** 1 Department of Reproduction Management, Leibniz Institute for Zoo and Wildlife Research, Berlin, Germany; 2 Laboratory of Embryology, Faculty of Veterinary Medicine, University of Teramo, Teramo, Italy; 3 Friedrich-Loeffler-Institut, Federal Research Institute for Animal Health, Institute of Molecular Pathogenesis, Jena, Germany; 4 Paris Zoo, Paris, France; 5 Conservation Medicine Services, Potters Bar, Hertfordshire, United Kingdom; 6 Garston Veterinary Group, Frome, United Kingdom; Hebrew University, ISRAEL

## Abstract

Tuberculosis (TB) occurs in a wide range of mammalian species and thus poses a health risk to humans living or working in close proximity with TB infected animals. Despite a high incidence of *M*. *bovis* infections in domestic or wildlife species tuberculosis infections in rhinoceros have so far been very limited. Over the past 53 years, tuberculosis of the respiratory tract has been confirmed in just 22 rhinoceros, most of those infected not by *M*. *bovis* but *M*. *tuberculosis*. However, because of the zoonotic risk TB testing is recommended or becomes even mandatory in endangered species. The dilemma in rhinoceros and many other wildlife species; non-validated tests are highly inconsistent in their ability to identify TB infection. Current lack of TB diagnostics may result in TB positive rhinoceros living with the infection, transmitting it to those around them or in euthanasia of animals found unconfirmed at necropsy. This is an unacceptable diagnostic status considering that some species are critically endangered and therefore should not be euthanized in order to confirm suspicion of disease. To overcome this shortcoming we used bronchoscopy to detect mycobacteria in respiratory fluids of TB suspicious rhinoceros. Fluids from seven, TB suspicious white rhinoceros were harvested during 21 bronchoscopies. Our new approach: In addition to bacterial culture a dual quantitative PCR system tested for the general presence of DNA from NTM and more specifically for DNA from MTC. Both, bacterial culture and qPCR were negative for MTC in respiratory fluids of all rhinoceros (7/7). At the same time, respiratory fluids from six rhinoceros tested positive for the presence of NTM or other closely related bacteria (6/7). *M*. *tuberculosis* was found only once in an oesophageal aspirate. The high incidence of mycobacterial DNA in the respiratory tract suggests that white rhinoceros, as strict grazers, are immensely exposed to environmental bacteria of this genus. Presence of NTM in the respiratory or intestinal system could possibly cause false positive results in intradermal tests. A wider use of bronchoalveolar lavage is warranted to further elucidate immunologic response to NTM and exposure to, incidence and prevalence of MTC infections in rhinoceros.

## Introduction

Tuberculosis (TB), caused by various members of the *Mycobacterium* (*M*.) *tuberculosis* complex (MTC) belonging to the genus *Mycobacterium*, occurs in a wide range of mammalian species with considerable species-specific variation in their susceptibility to contract and develop long term sub-clinical disease or to develop clinically relevant disease. Mycobacteria other than tuberculosis, also called non-tuberculous mycobacteria (NTM) may, in rare circumstances, cause disease in mammalian hosts but could be associated with typical tuberculous disease found in non-mammalian vertebrate species, e. g. in birds (e. g. *M*. *avium* complex) or fish (e. g. *M*. *marinum*). Control of tuberculosis in livestock relies to a large part on test-and-slaughter approach. In regions where livestock comes in contact with wildlife, a risk of bidirectional spill over is a real and pressing problem. For instance the African buffalo population of South Africa became infected by *M*. *bovis* through its interaction with infected domestic cattle [1,2]. The buffalos are now acting as maintenance hosts and spread the disease to carnivores feeding on them as well as to other species with which they come in contact. To date a large number of wildlife species have tested positive for *M*. *bovis* including southern black rhinoceros (*Diceros bicornis minor*), African buffalo (*Syncerus caffer*), chacma baboon (*Papio ursinus*), lion (*Panthera leo*), cheetah (*Acinonyx jubatus*), greater kudu (*Tragelaphus strepsiceros*), leopard (*Panthera pardus*), spotted hyena (*Crocuta crocuta*), large-spotted genet (*Genetta tigrina*), warthog (*Phacochoerus aethiopicus*), bushpig (*Potamochoerus porcus*), and eland (*Taurotragus oryx*) [1,2,3,4,5].

Tuberculosis in rhinoceros species has so far been an incidental, sporadic finding at post mortem surveillance suggesting either a general, low sensitivity of these species to this disease or TB as an under-recognized threat [6]. Over the last 53 years, there have been just 16 reports from 4 continents on 22 incidental cases of tuberculosis (Table 1). In all reports on MTC infections in rhinoceros the pulmonary system was the primary infected organ [6,7]. The majority, 15 cases were *M*. *tuberculosis* infections (15/21), 12 of those in black (*Rhinoceros bicornis*), 2 in white (*Ceratotherium simum*) and one in an Indian rhinoceros (*Rhinoceros unicornis*). Seven cases reported on infections caused by *M*. *bovis*, thereof, five in black and two in white rhinoceros (Table 1) [3,8,9,10,11,12,13,14,15,16,17]. In attempts to treat animals suspicious of *M*. *tuberculosis* five rhinoceros received antibiotic treatment [18,19,20]. Yet, only one was treated successfully [18]. Overall, incidental MTC findings in rhinoceros are in contrast to the high incidence of *M*. *bovis* in wildlife populations e.g. in southern Africa. Rhinoceros, exposed to excessively *M*. *bovis*-infected buffalo populations (up to 40%) [21] never developed a manifestation of this endemic disease [1,22,23].

**Table 1:** List of publications on tuberculosis in rhinoceros ordered by species and type of mycobacterium diagnosed.

Rhinoceros species		Number of	Reference
	Detected species of the *Mycobacterium tuberculosis* complex (MTC)	infected animals	
Black rhinoceros	*M*. *tuberculosis*	1	Takagi et al., 1964
Black rhinoceros	*M*. *tuberculosis*	1	Powers et al., 1967
Black rhinoceros	*M*. *tuberculosis*	1	Godfrey et al., 1990
Black rhinoceros	*M*. *tuberculosis*	1	Barbiers et al., 1994
Black rhinoceros	*M*. *tuberculosis*	2	Valandikar et al., 1996
Black rhinoceros	*M*. *tuberculosis*	2	Oh et al., 2002
Black rhinoceros	*M*. *tuberculosis*	2	Ball et al., 2007
Black rhinoceros	*M*. *tuberculosis*	1	Duncan et al., 2009
Black rhinoceros	*M*. *tuberculosis*	1	Miller et al., 2015
Black rhinoceros	*M*. *bovis*	1	Keep and Basson, 1973
Black rhinoceros	*M*. *bovis*	2	Mann et al., 1981
Black rhinoceros	*M*. *bovis*	1	Epsie et al., 2009
Black rhinoceros	*M*. *bovis*	1	Miller et al., 2017
White rhinoceros	*M*. *tuberculosis*	2	Karlstam et al., 2015
White rhinoceros	*M*. *bovis*	1	Stetter et al., 1995
White rhinoceros	*M*. *bovis*	1	Dalvisio et al., 1992
Greater one-horned rhinoceros	*M*. *tuberculosis*	1	Thapa et al., 2016

Because of the risk captive infected animals pose to their environment [17] and to all those who come in contact with them [16,24,25], TB testing of captive animals has been recommended [26]. Testing, however, is not such a trivial matter in rhinoceros. A variety of tests have been applied in rhinoceroses including ELISA [8,12,20], intradermal tuberculin test [12,26], nasal wash [24,27] or tracheal/gastric lavage [28] followed by bacterial culture, serological tests including multi-antigen print immunoassay (MAPIA) and ElephantTB STAT-PAK assay [18,20], DPP VetTB assay (unpulished data, Cracknell, Chembio Diagnostic systems, NY, USA) and rhino-specific interferon gamma (IFN-γ) assay [8]. Intradermal tuberculin tests (PPD) using >100 purified proteins for testing seem to give inconclusive results in rhinoceros [12,26,27]. The large portfolio of antigens in intradermal tuberculin tests may be reason for frequent cross reactivity when used in new species and explains the uncertain diagnostic value of this test in rhinoceros. At times, injection at different sites on the same animal would yield different outcomes. The other tests seem to be similarly inconsistent in their ability to correctly identify infection or its absence. In a North American zoo survey, none of 53 tested rhinoceros from three different species produced a clear positive result in intradermal tuberculin test [26]. In one white rhinoceros euthanized following an equivocal intradermal testing, post mortem tests were negative for mycobacteria [26]. A negative intradermal tuberculin test in one black rhinoceros was contradicted when *M*. *tuberculosis* was found at post mortem. Such false-negative result could possibly represent a subset of TB infected animals anergic to purified protein derivative (PPD) used for the intradermal test [29]. Consequently, it was concluded that intradermal tuberculin test is not recommendable for TB diagnosis in rhinoceros unless a species-specific test is developed [26]. Other studies in white (*Ceratotherium simum simum*) and black rhinoceros (*Diceros bicornis*) showed inconsistent results in serological and intradermal tuberculin tests [12,27]. One of these animals positive on culture of nasal wash was confirmed positive at necropsy. Another negative on nasal wash was positive at necropsy [27]. Three other black rhinoceros were positive on intradermal test and *M*. *bovis* specific serum ELISA yet, negative at necropsy [20].

Authorities in some countries mandate TB testing of every species which was in close proximity of a TB positive specimen including rhinoceroses. However, this creates the stalemate situation, where the current lack of TB diagnostics in rhinoceros makes it difficult to diagnose animals as disease free lifting any TB suspicion.A rhino-specific IFN-γ assay is presently under development for TB diagnosis in rhinocerotidae. The assay was determined to have specificity (correctly identifying healthy animals as such) of 94%. Sensitivity (correctly identifying diseased animals) is still to be determined [8]. With lack of reliable and consistent TB testing technique, some rhinoceroses go on living with the infection, and transmitting it to those around them. Others are euthanized based on test results that may not be conclusively accurate but are not confirmed at necropsy [20,26]. Both alternatives are far from being ideal. For the sake of captive rhinoceros, the animals in neighbouring exhibits, which might be more susceptible to the disease, and the people around (attendants and the general public) it is imperative to work on improving the accuracy and reliability of the diagnostic tests in rhinocerotidae.

Culture is still recognised as the ‘gold standard’ for tuberculosis diagnosis. In rhinoceros, and other charismatic megafauna, obtaining clinically relevant samples TB diagnostic is extremely challenging. The aim of this study was to develop a method of obtaining clinically relevant samples for bacterial culture, using a repeatable and consistent method that can be applied for the diagnosis of tuberculosis in any of the rhinoceros species, and to outline the novel methods of tuberculosis case confirmation in the samples obtained. For this reason, bacterial culture of the samples was combined with two quantitative PCRs: one qPCR targeted DNA of MTC, and the other targeting DNA from all mycobacterial species or other closely related bacteria. This dual molecular testing was at the core of this new diagnostic approach for tuberculosis in rhinoceros. Molecular detection of bacterial DNA is fast, specific, sensitive and not vulnerable to be spoiled by possible bacterial or fungal overgrowth during culture. Pathogens may be detected even if they were not able to multiply in culture for whatever reason.

In this study we included animals from facilities, TB had been diagnosed in other ungulate species and intradermal and serological tests had suggested a TB infection. Our aim was to replace controversial intradermal and serological tests in captive rhinoceros under TB suspicion by implementing bronchoscopy and lung lavage, as a more efficient but safe TB diagnostic tool for rhinoceros. Bronchoalveolar lavage aimed at detecting the presence of MTC, NTM or closely related bacteria or their DNA in respiratory fluids of rhinoceros, in which TB had been suspected based on other non-validated tests.

## Materials and methods

### Ethics statement

This study was conducted on captive white rhinoceros under suspicion of active tuberculosis infection. The study was carried out in strict accordance with the German National Protection of Animals Act from 24.07.1972 and its last revision from 15th July 2009. Under this Act, an examination directed towards diagnosing an animal’s disease is not defined as an animal experiment (§7) but as a mandatory act of animal welfare. Although not mandated by law, the study on diagnosing TB in rhinoceros was also approved by IACUC animal ethics committee of the Leibniz Institute for Zoo and Wildlife Research (Permit number: 2014-09-04).

### Animals

The bronchoalveolar lavage (BAL) and supplemental lavage of the oesophagus (OL) and air sacs were performed in 7 white rhinoceros (*Ceratotherium simum simum*: n *=* 2 males, n = 5 females) aged between 5–21 years in two European zoos. In both institutions other ungulates on the same exhibit as the rhinoceros had been diagnosed positive for *M*. *bovis* at necropsy. Four of seven rhinoceros tested positive for MTC in serological tests performed prior to the bronchoscopy (Table 2). All of those animals were clinically healthy without any signs of respiratory disease or chronic wasting. To further diagnose the presence or absence of mycobacterial infection, BAL and OL were performed 2–4 times in 8–24 months intervals. One female, being negative in both serum and first BAL, was not tested again due to advanced age and related sedation concerns.

**Table 2:** Serological and intradermal tuberculin test results in seven white rhinoceros prior to bronchoalveolar lavage.

white rhinoceros	DPP serum test	Intra dermal
	(non validated)	Skin test
1	***M bovis* positive**	positive
2	*M* ***bovis* positive**	negative
3	**negative**	negative
4	**negative**	negative
5	***M tuberculsosis* positive**	negative
6	*M* ***tuberculsosis* positive**	negative
7	negative	negative

### Sedation

To allow the passing of the endoscope through the nasal cavity and the larynx into the trachea, all animals received a standing sedation. Sedation was achieved by intramuscular injection of 27 ± 0.5 mg detomidine hydrochloride (Domidine 10 mg/mL, Eurovet Animal Health B.V., Bladel, The Netherlands), 27 ± 0.5 mg butorphanol (Torbugesic Vet 10 mg/mL, Zoetis B.V., Capelle a/d IJssel, The Netherlands), and 94 ± 7 mg Ketamine hydrochloride (Ketamin 10% WDT, Henry Schein VET GmbH, Hamburg, Germany) based on estimated body weight of 1,500–2,000 kg. When needed, sedation was maintained by i.v. administration of additional 2–10 mg of detomedine hydrochloride and butorphanol each and 50–100 mg of ketamine into the ear vein. Sedation was antagonised by administration of 207 ± 5 mg naltrexone hydrochloride (Trexonil, Wildlife Pharmaceuticals (PTY) Ltd., White River, South Africa) and 41 ± 0,5 mg atipamezole hydrochloride (Atipam 5 mg/mL, Eurovet Animal Health B.V.). Half the reversal was given i.m. and the other half was given i.v. Animals were normal and alert two to three minutes after the antagonist was given. Total time for the procedure from first injection to full recovery of the animal was less than 45 minutes. Considering a 25 minute sedation induction time, the bronchoscopy and lavage, the core procedure itself took only 20 minutes.

### Bronchoalveolar and oesophageal lavage

Following sedation, a flexible 3.5 m video-chip endoscope (ESO Jürgen Ohle Endoskopie Technik und Chirurgiemechanik, Wedel, Germany) connected to a mobile video processor and monitor (ESO Jürgen Ohle Endoskopie Technik und Chirurgiemechanik) was introduced into one of the nostrils. Through the ventral conchae the endoscope was advanced through the rostrum to visualize the larynx before further advancing it into the trachea. Under direct endoscopic view of the carina and the bronchi a disposable 6 m catheter (Gynetics Medical Products N.V., Lommel, Belgium) was advanced into at least three bronchi of the left and the right lung lobes, respectively. Over the course of the procedure a total of 100 mL of 0.9% NaCl solution were instilled in different bronchi. The fluids were then aspirated into a sterile 50-mL Falcon tube using a portable suction pump (DC15 Endo, Asskea Medical, Greussen, Germany). After collection of bronchoalveolar samples the endoscope was retracted to the larynx, the inner catheter replaced and then advanced into the oesophagus. In the oesophagus 30–50 mL of 0.9% NaCl were instilled and the fluid was aspirated thereafter again by use of the suction pump. Fluids aspirated from the lung and oesophagus were collected in separate Falcon tubes. In addition to samples from the lung and oesophagus, few opportunistic samples were collected from the dorsal air sacs when these showed signs of infection such as hyperaemic mucosa, swollen lymphatic tissue or purulent mucus. Samples collected from different bronchi of both lungs were combined and kept separate from samples from the oesophagus. All samples were sealed in sterile zip-lock bags for additional protection and shipped within 48 h at 4°, without any further handling, to the Friedrich-Loeffler-Institut (FLI, Jena, Germany) for TB testing.

### Sample processing and bacterial culture

All samples were tested for the presence of mycobacteria by bacterial culture and real-time PCR for members of the MTC, NTM or other closely related bacteria. Briefly, 10 mL of the liquid samples collected from the rhinoceros respiratory system were centrifuged at 3,800 g for 20 min at 10°C. The sediments were re-suspended with equal volumes (10 mL) of PBS and NALC (N-acetyl-L-cysteine-NaOH; final NaOH concentration: 1%) and incubated at room temperature with gentle shaking for 25 min. After adding 20 mL of PBS, it was mixed thoroughly and centrifuged at 3,800 g for 20 min. The sediment was washed again with PBS. The final sediment was re-suspended with 1.5 mL PBS, wherefrom 500 μL were removed for direct DNA extraction. Antibiotics mixture (100 μL) containing polymyxin B, amphotericin B, nalidixic acid, trimethoprim, and azlocillin (PANTA) (Becton Dickinson, Heidelberg, Germany) was added to the remaining 1.0 mL of suspension. Two solid growth media (i) Stonebrink medium with pyruvate and antibiotics mixture containing polymyxin B, amphotericin B, carbenicillin and trimethoprim (PACT) (Bioservice Waldenburg, Waldenburg, Germany), and (ii) Löwenstein-Jensen medium with glycerol and PACT (Artelt-Enclit, Borna, Germany) were inoculated with 150 μL of the suspension. Additionally, one liquid medium (Kirchner bouillon; Artelt-Enclit, Borna, Gemany) was also inoculated with 150 μL. Incubation was performed at 37°C for at least 12 weeks.

### Molecular detection of mycobacterial DNA

Molecular detection of mycobacterial DNA was done twice: First on fresh lavage samples prior to the bacterial culture and second, after the bacterial culture had ended, 12 weeks later (Table 3). For extraction of DNA directly from wash samples, the separated 500 μL-portions (see above) were centrifuged at 13,000 g for 10 min and the sediments were processed using DNeasy Blood and Tissue kit (Qiagen, Hilden, Germany) following the manufacturer’s instructions. Subsequently, DNA extracts were subjected to the MTC-qPCR system consisting of two separate MTC-specific qPCRs, with the insertion sequence (IS) *1081* and a hypothetical helicase as targets [30]. Secondly, DNA extracts were subjected to a qPCR targeting the mycobacterial 16S rRNA gene, to detect all members of the genus *Mycobacterium* (MG) (S1 Table) [31]. All three qPCR assays were performed as duplex PCRs co-amplifying the eukaryotic beta-actin DNA fragment as internal control (IC) [30]. The qPCRs were performed using the TaqMan Gene Expression Master Mix (Fischer Scientific GmbH, Schwerte, Germany) and run on an Applied Biosystems 7500 Real-Time PCR System (Applied Biosystems, Darmstadt, Germany). Sequences of the used primers and probes are listed in the S1 Table. The thermal cycling started with a denaturation step (15 min at 95°C), followed by 45 amplification cycles with 95°C for 60 sec (MTC)/15 sec (NTM), 60°C for 30 sec (MTC)/60 sec (NTM), and 72°C for 30 sec [30]. With respect to the susceptibility of sample contamination, the cut-off threshold cycle Ct value was set at 36.

**Table 3:** Results of bacterial culture and PCRs of respiratory and oesophageal aspirates from white rhinoceros for diagnosing bacteria of the MTC.

White	Procedure	Sampling	Lavage	Results of bacterial	MTC-qPCR Heli/IS1081**	conventional MTC-PCR*	MG-qPCR 16S rRNA
rhinoceros no.	no.	date	type	culture	[direct DNA extraction]	[cultured bacteria]	direct DNA extraction
1	1	07.10.2014	broncho alveolar	negative	negative	negative	n.d.
1	2	06.05.2015	broncho alveolar	negative	negative	negative	negative
1	3	15.05.2017	broncho alveolar	negative	negative	negative	inconclusive
1	4	30.10.2017	broncho alveolar	negative	negative	negative	positive
2	5	07.10.2014	broncho alveolar	negative	negative	negative	n.d.
2	6	06.05.2015	broncho alveolar	negative	negative	negative	positive
2	7	15.05.2017	broncho alveolar	negative	negative	negative	positive
2	8	30.10.2017	broncho alveolar	negative	negative	negative	positive
3	9	07.10.2014	broncho alveolar	negative	negative	negative	n.d.
3	10	06.05.2015	broncho alveolar	negative	negative	negative	negative
3	11	15.05.2017	broncho alveolar	negative	negative	negative	inconclusive
3	12	30.10.2017	broncho alveolar	negative	negative	negative	positive
4	13	07.10.2014	broncho alveolar	negative	negative	negative	n.d.
4	14	06.05.2015	broncho alveolar	negative	negative	negative	negative
4	15	15.05.2017	broncho alveolar	negative	negative	negative	positive
4	16	30.10.2017	broncho alveolar	negative	negative	negative	positive
5	17	05.11.2014	broncho alveolar	negative	negative	negative	negative
6	18	05.11.2014	broncho alveolar	negative	negative	negative	negative
6	19	26.06.2015	broncho alveolar	negative	negative	negative	negative
7	20	05.11.2014	broncho alveolar	negative	negative	negative	inconclusive
7	21	26.06.2015	broncho alveolar	negative	negative	negative	negative
White	procedure	Date	Supplemental	Bacterial	MTC-qPCR	MTC-PCR	MG-PCR
rhinoceros	(#)		samples	culture	Heli/IS1081**	Heli*	16S (Ct)
1	1	07.10.2014	oesophageal	negative	n.d.	negative	positive
1	2	06.05.2015	oesophageal	negative	n.d.	negative	positive
2	4	07.10.2014	oesophageal	negative	n.d.	negative	positive
2	5	06.05.2015	oesophageal	negative	n.d.	negative	positive
3	7	07.10.2014	oesophageal	negative	n.d.	negative	negative
3	8	06.05.2015	oesophageal	negative	n.d.	negative	positive
4	10	07.10.2014	oesophageal	negative	n.d.	negative	negative
4	11	06.05.2015	oesophageal	negative	n.d.	negative	positive
4	11	06.05.2015	Air sac	negative	n.d.	negative	positive
5	13	05.11.2014	oesophageal	*Rhodococcus* ssp.	negative	negative	positive
6	14	05.11.2014	oesophageal	*M*. *tuberculosis* Manu 2	positive	n. d.	positive
6	15	26.06.2015	oesophageal	negative	n.d.	negative	positive
7	16	05.11.2014	oesophageal	negative	n.d.	negative	positive
7	16	05.11.2014	Air sac	negative	n.d.	negative	positive
7	17	26.06.2015	oesophageal	negative	n.d.	negative	positive

*Conventional MTC-PCR targeting the gene of a hypothetical helicase (Heli), specific for bacteria of the MTC

**qPCR targeting Heli and IS*1081*, specific for bacteria of the MTC

MTC = *M*. *tuberculosis* complex. n.d. = not determined.

### Molecular identification of bacterial isolates

DNA from cultivated isolates was extracted by suspending colony material in 100 μL sterile water, heat-inactivating the bacteria at 80°C for 20 min, ultra-sonicating (35 kHz), boiling the suspension for 10 min, and then centrifuging (13,000 g, 5 min). The supernatant containing the DNA was harvested and centrifuged to remove debris. The final supernatant containing the DNA extract was then subjected to a conventional PCR for identification of the genus *Mycobacterium* based on 16S rRNA analysis [16]. If mycobacterial DNA was detected species identification was done by conventional PCRs targeting the IS *1245* and *901* for members of the *M*. *avium* complex [32,33] or an hypothetical helicase [34,35] for MTC members. DNA of mycobacterial species not identifiable by these PCRs were identified by nucleotide sequence analysis GATC, Konstanz, Germany) of a PCR generated DNA fragment of the 16S rRNA gene [31].

### Spoligotyping of mycobacteria

Spoligotyping was performed with DNA extract generated from culture material identified as MTC-positive by MTC-specific qPCR (see above) using a microarray system (Alere Technologies, Jena, Germany) with integrated data analysis [36]. Spacer sequences 1 to 43 of the direct repeat (DR) region of the MTC genome were analysed.

## Results

**Fig 1:**
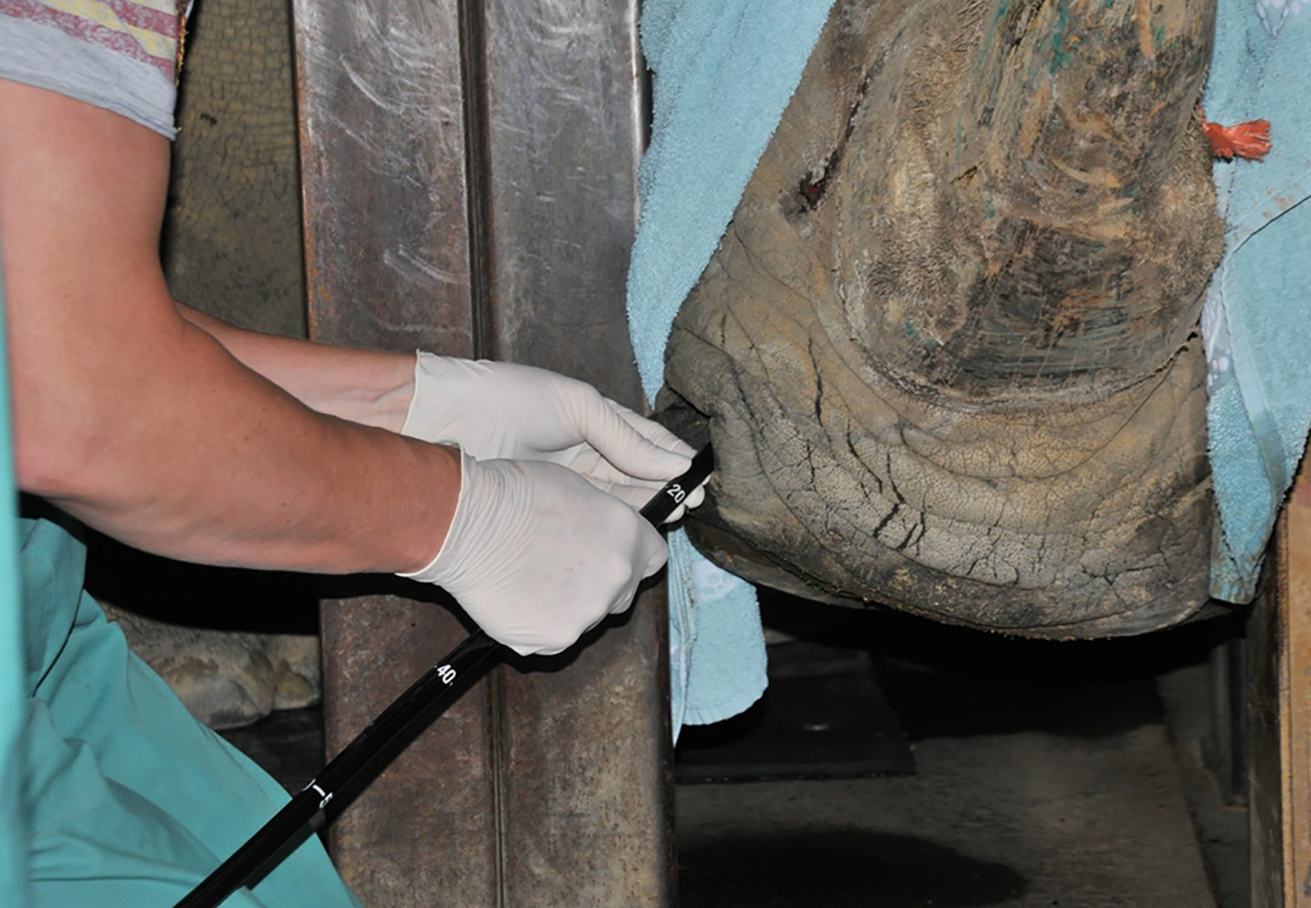
Sedated white rhinoceros with endoscope inserted into the left nostril.

**Fig 2:**
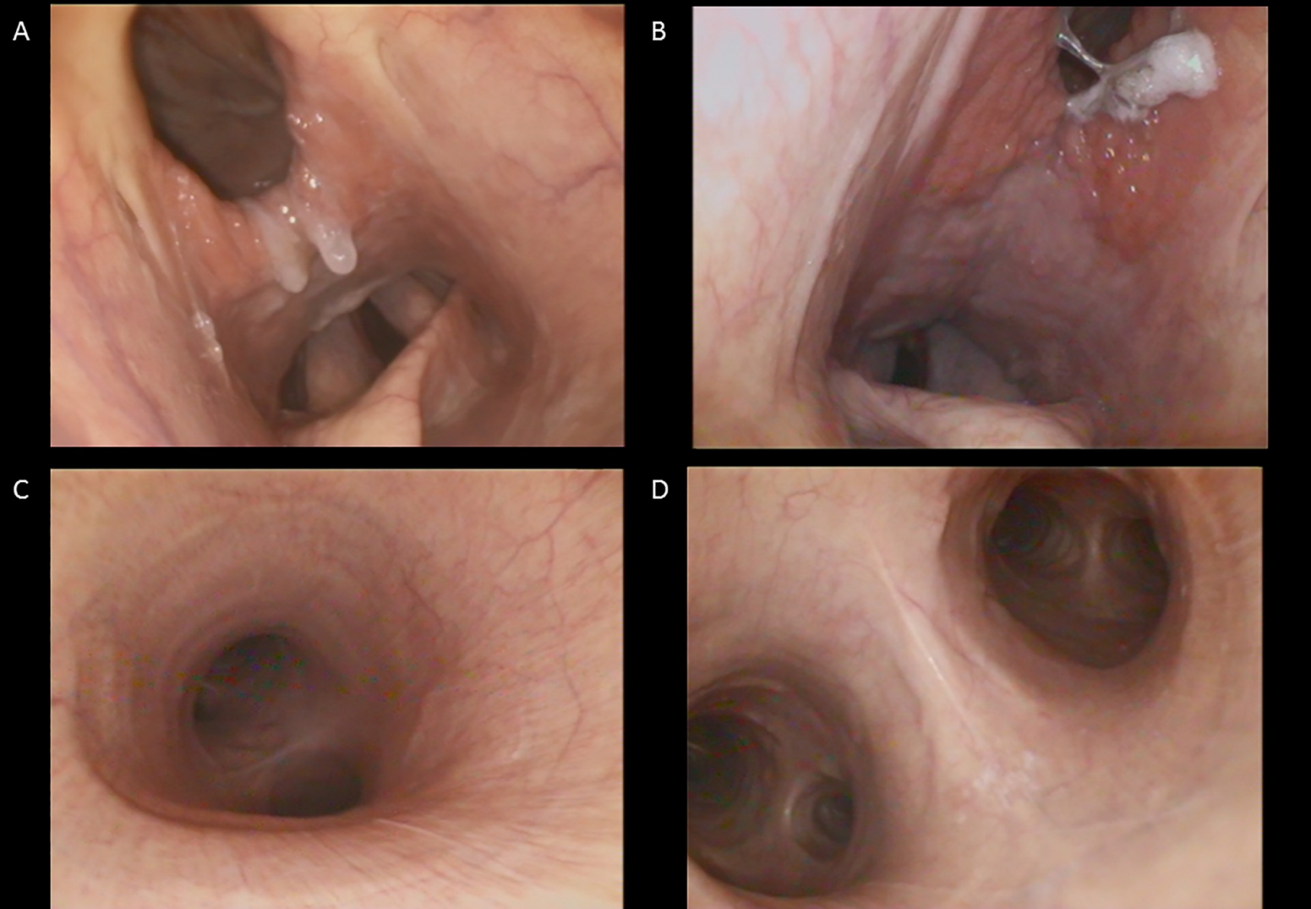
A-D endoscopic views of the respiratory tract in the white rhinoceros. A: View of the epiglottis and rima vocalis below the opening to the dorsal air sac. B: View of the epiglottis and rima vocalis. Swollen lymphatic tissue surrounds the opening of the dorsal air sac. C: View of the trachea, carina tracheae and two main bronchi separating left from right lung. D: View onto the carina tracheae and bifurcatio into smaller bronchi or each lung.

Flexible endoscopy facilitated access to and visualization of the larynx, trachea, major bronchi, air sacs and oesophagus for lavage in all seven rhinoceros (Figs 1 and 2). The distance covered from the nostril to the carina and large bronchi varied between 1.8–2.3 m. The trachea and bronchi showed no indication for acute or chronic infection in any of 21 bronchoscopies. Frequently observed, superficial infections of the larynx or air sacs were sampled but not associated with MTC infection (Fig 2).

Bronchoalveolar fluids were aspirated from all seven rhinoceros during all 45 minute procedures (21/21). Aspirates were turbid and rich in mucus from the respiratory system. Bronchoalveolar, air sac and oesophageal aspirates were investigated by bacterial culture and the dual qPCR for the presence of mycobacteria or mycobacterial DNA, respectively. The dual qPCR system tested firstly specifically for the presence of MTC-DNA and secondly for the presence of mycobacterial DNA in general including DNA of other bacterial taxa closely related to the genus *Mycobacterium*.

**Fig 3:**
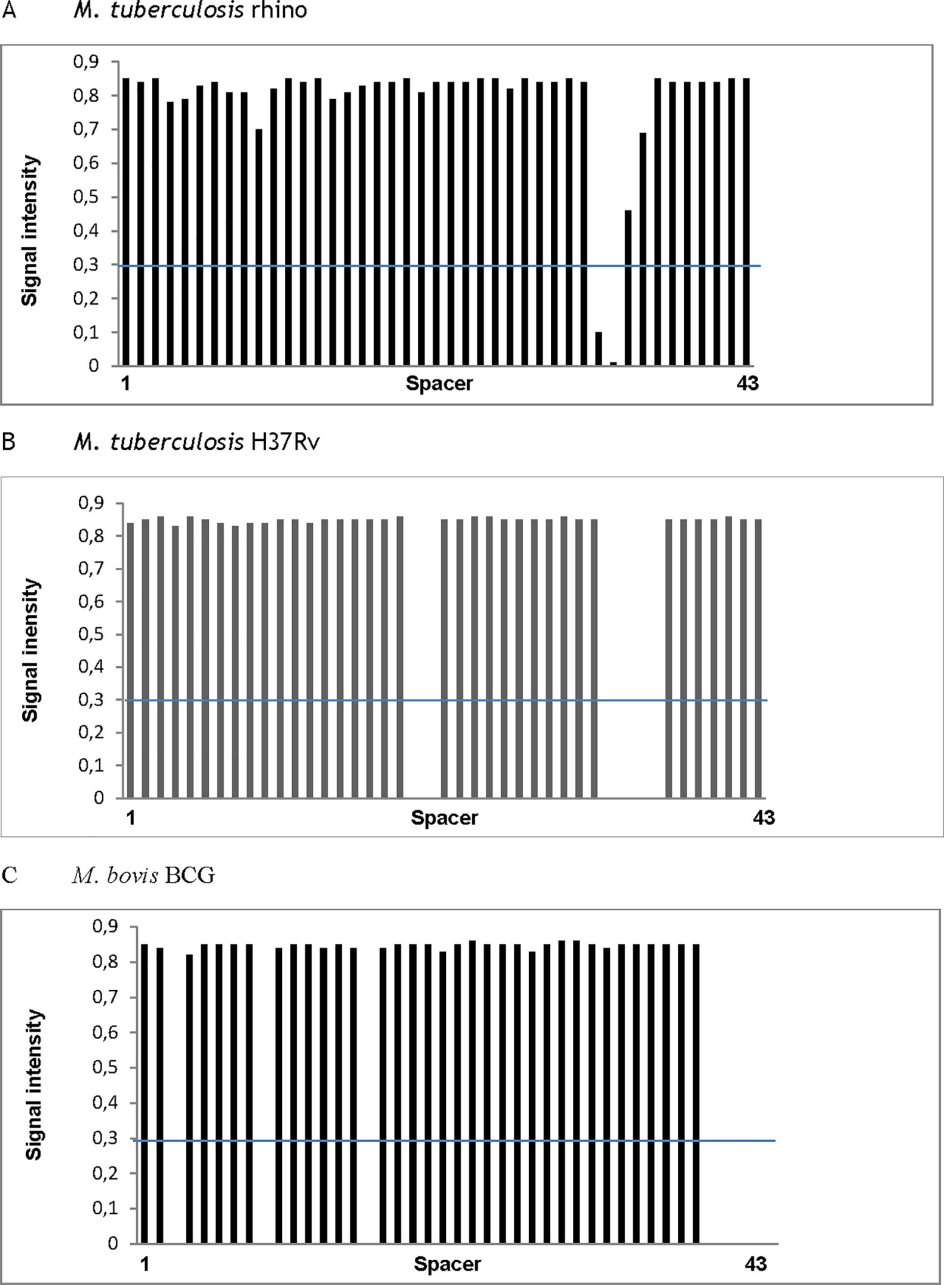
Spoligotyping patterns of the *M*. *tuberculosis* isolate detected in the oesophageal aspirate of rhinoceros 6 (A) and two MTC control strains (B: *M*. *tuberculosis* H37Rv; C: *M*. *bovis* BCG). blue horizontal line: cut off level.

Positive serological and intradermal tuberculin tests had suggested an immune response to MTC in four rhinoceros (n = 4/7, Table 2). Yet, all fluids from the lower and upper respiratory tract (21/21) were negative for the presence of MTC or their DNA (Table 3). While all samples from the respiratory system were negative for MTC, oesophageal samples from two animals showed bacterial growth and positive qPCR after 3 months of culture. In one rhinoceros, very weak growth of *M*. *tuberculosis* in the liquid growth medium was detected in the oesophageal sample. Spoligotyping further identified this *M*. *tuberculosis* isolate being from the MANU 2 genotype (Fig 3). In a second animal, *Rhodococcus* sp., which belongs to the order of Corynebacteriales just as the Mycobacteriaceae, was isolated from the oesophageal lavage. However, subsequent oesophagus samples taken at follow-up examinations remained negative for MTC and *Rhodococcus* sp. in both animals (Table 3).

DNA of non-tuberculous mycobacteria or closely related bacteria was detected in the majority of all collected fluids from the respiratory system and oesophagus. Overall, 41% (7/17) of the bronchoalveolar and 76% (13/17) of the air sac and oesophageal fluids contained DNA from bacteria of the genus *Mycobacterium* (Table 3). Fluids from the respiratory system tested positive for the presence of DNA from NTM at least once in four out of seven animals.

## Discussion

Bronchoscopy and bronchoalveolar lavage has to date been greatly underutilised for the inspection of and sampling from the respiratory system from tuberculosis suspicious rhinoceros. Forty-five minute standing sedation, of which the intervention itself just took 20 minutes, demonstrated the practicability of bronchoscopy in rhinoceros using regular, horse endoscope equipment. In this study, bronchoscopy facilitated reliable access to and lavage of different bronchi in the lung, the primary organ infected by MTC in rhinoceros [6,8]. Standing sedation facilitated physiological pulmonary function and therefore probably delivered a fairly good representative sample of alveolar exposure. The use of molecular detection of mycobacterial DNA immediately after the collection and prior to start of the 12 week standard bacterial culture provided a fast, instantaneous result on whether any animal suffered from an active TB infection and was shedding bacteria. Such instant, preliminary qPCR result without 3 months of diagnostic delay due to bacterial culture limitations has huge benefits on the decision whether or not to start immediate antibiotic therapy in rhinoceros under TB suspicion. However, bacterial culture and qPCR failed to detect mycobacteria of the *M*. *tuberculosis* complex or their DNA in any of the aspirated, respiratory fluids from TB suspicious rhinoceros. Strikingly, at the same time, respiratory fluids from four out of seven animals tested positive for the presence of genus-specific, non-tuberculous mycobacterial DNA. Moreover, samples from the air sacs or the oesophagus tested positive for the presence of NTM DNA in all animals. This high incidence of NTM DNA in both, the respiratory tract and the oesophagus, suggests that the immune system of white rhinoceros, as strict grazers, is immensely exposed to environmental mycobacteria. Exposure to and presence of NTM in the respiratory or intestinal system could possibly cause false positive results specifically with non-validated, intradermal tuberculin tests.

In principle, rhinoceros are susceptible to infections of bacteria of the MTC [6]. Yet, despite sharing the same habitat with domestic and wildlife species known to host this disease [37], reports on tuberculosis in wild or captive rhinoceros have been scarce. To date just 22, mostly captive rhinoceros of three different species from four continents have been reported with confirmed MTC infections of the respiratory system (summarized in Table 1) [6]. The majority of those have been black rhinoceros infected by *M*. *tuberculosis*, which is supposedly transmitted from humans. To further elucidate susceptibility of white rhinoceros to endemic tuberculosis three white rhinoceros have been experimentally infected with a virulent *M*. *bovis* isolate in a recent study [28]. During the 20 months trial period none of the animals developed clinical disease. No mycobacteria were found in lungs or lymph nodes post mortem but *M*. *bovis* genetic material was detected by PCR suggesting that healthy young white rhinoceros maybe resistant to development of *M*. *bovis* disease [28].

Regardless of the low incidence of tuberculosis in rhinoceros and their low susceptibility to it, the possible exposure to MTC in natural habitats or in captive collections demands for an easy, reliable and safe diagnostic [38]. Furthermore, the zoonotic risk for zoo personnel and the public demands for mandatory tuberculosis testing in animals which have been exposed to MTC, regardless of their conservation status [17,38, 39]. The dilemma in rhinoceros; commercial tests are inconsistent in their ability to identify tuberculosis infection or its absence [6]. Possible cross-reactivity of commercial serological diagnostic tests with the rhinoceros’ immune response to non-tuberculous mycobacteria (NTM) and subsequent risk of false positive results are of great concern [6]. Our MG-qPCR results on the ‘ubiquitous’ presence of DNA from bacteria of the Mycobacterium genus in white rhinoceros respiratory organs further supports this concern.

In general, TB diagnosis other than post mortem is problematic in wildlife species as it requires the development of a sensitive and specific TB test in each species. This requires the characterization of serum reactive antigens, their specific antibodies or increase in IFN-γ either in experimentally infected animals or in retrospective studies of known positive animals [8, 40,41]. In nonhuman primates, such specifically developed serodiagnostic TB test showed 90% sensitivity and 99% specificity without any cross reactivity to NTM [41]. However, sero-reactive antigens may vary between species and the type of mycobacterial infection. Experimental infection or retrospect serum study in known-positive animals is difficult to accomplish in highly endangered species as the rhinoceros. Therefore, great hope lays on a new, experimentally developed IFN-γ assay with high sensitivity but not determined specificity [8].

Positive bacterial cultures from the oesophagus of two animals were identified as *Rhodococcus*, an acid-resistant genus, closely related to mycobacteria in one animal, and as *M*. *tuberculosis* in the other. In both cases the qPCR test systems, targeting MTC-specific as well as genus specific DNA, confirmed these results and demonstrated the accuracy of molecular species identification. Despite imminent qPCR testing of the samples prior bacterial culture MTC-specific genes were found only after 12 weeks of culture. Because, the *M*. *tuberculosis* or its DNA was not found again on successive lavages shortly after the positive finding, the animal was not treated.

These results do not indicate disease in these animals but they show that the presence of mycobacteria including MTC can be detected by this procedure on a very low level if they are present in the respective compartment at the time of sampling. The detection of *M*. *tuberculosis* in one animal underlines this fact. This result might be regarded as a fortunate finding at the moment of sampling and the failure to reproduce it by follow-up examination does not characterize it as false result.

Repeated BAL may represent the best feasible diagnostic in rhinoceros to detect active TB infection and to distinguish from false positive, intradermal or immunological tuberculosis tests. However, even if BAL failed to detect evidence of active respiratory tuberculosis, a recent, in-depth study in bacterial culture negative white rhinoceros described the presence of minor, non-visible tuberculosis lesions in the lung post mortem pointing at a remaining risk for inactive tuberculosis [27,42], supposedly difficult to access even by broncho lavage. Thus, single BAL may not prevent from the possibility of intermittent shedding and risk of false negative results in the presence of a true but inactive TB infection. We tried to encounter this diagnostic gap by repeated BAL in standing position, from many different bronchi. This ensured that different lobes and bronchi were sampled each time, thus achieving a random sample set from the entire lung. However, the remaining pulmonary dead space in rhinoceros which remained not sampled is considerable, leaving the BAL with remote risk of providing a false negative result.

In conclusion, in vivo diagnosis of tuberculosis in wildlife species suffers from the absence of validated intradermal, immunological tuberculosis tests. Here, bacterial culture and qPCR of respiratory fluids from TB suspicious rhinoceros revealed high incidence of DNA from NTM in the respiratory system thus not confirming active tuberculosis in any of the TB suspicious animals. Therefore, we recommend the extensive use of bronchoalveolar lavage with subsequent bacterial culture and qPCR for the presence of NTM and MTC or their DNA in rhinoceros to diagnose shedding mycobacterial infection in live animals under TB suspicion. Immediate molecular detection of mycobacterial DNA in lavage fluids by qPCR adds a potent tool in wildlife Tb diagnostics as it provides, a short term diagnosis on possibly TB infected, actively shedding animals. Such immediate result has positive implications on better decision making on immediate TB treatment, husbandry, animal and staff management or euthanasia especially. Bronchoscopy might not be practical on large scale under free range conditions, but might aid in determining the prevalence of live shedding animals. More research and wider use of bronchoalveolar lavage is warranted to further elucidate exposure to, incidence and prevalence of *M*. *tuberculosis* and *M*. *bovis* infections in rhinoceros or other wildlife species for which tuberculosis tests are not established yet.

## Supporting information

S1 Table Used oligonucleotides and probes in the qPCR assays. (DOCX)
