# Supplementary material for: Differential detection of tuberculous and non-tuberculous mycobacteria by qPCR in lavage fluids of tuberculosis-suspicious white rhinoceros
Source: PLoS One. 2018 Nov 28;13(11):e0207365. doi: 10.1371/journal.pone.0207365 (PMC6261570; doi:10.1371/journal.pone.0207365)
Supplement: S1 Table — (DOCX) [file pone.0207365.s001.docx]

Supplemental Table S1: Used oligonucleotides and probes in the qPCR assays.

| qPCR | Oligo / Probe | DNA sequence (5' 🡪 3') | Final con­cen­tration in qPCR | Reference |
| --- | --- | --- | --- | --- |
| MTC Heli | MTC-HELI-4F MTC-HELI-4R MTC-HELI-4FAM | TTGATCAGGTCGACGATGTAG TCACCACCGACAAAGCGTC FAM-TCAACGACCCCAACGACTGGTGC-BHQ1 | 250 nM 250 nM 125 nM | Anony­mous, 2017 |
| MTC IS*1081* | MTC-IS1081-5F MTC-IS1081-5Rn MTC-IS1081-5FAM | CTCTCGACGTTCATCGCCG TGGCGGTAGCCGTTGCGC FAM-ATTGGACCGCTCATCGCTGCGTTC-BHQ1 | 250 nM 250 nM 125 nM | Anony­mous, 2017 |
| MG 16S rRNA | Mycobac16S_F Mycobac16S_R Mycobac16S_SFAM | TGCGGGCGA­TACGGGCAGRCT CCACACCTAGTWCCCACCGTT­TAC FAM-TGGCGAAGGCGGGTCTCTGGGCAGTA-BHQ1 | 50 nM 300 nM 200 nM | Nieter, 2016 |
| IC β-actin | ACT2-1030-F ACT-1135-R ACT-1081-1105-YAK | AGCGCAAGTACTCCGTGTG CGGACTCATCGTACTCCTGCTT YakimaYellow-TCGCTGTCCACCTTCCA­GCAG­ATGT-BHQ1 | 200 nM 200 nM 100 nM | Toussaint et al., 2007 |
